# Supplementary material for: Why laparoscopists may opt for three-dimensional view: a summary of the full HTA report on 3D versus 2D laparoscopy by S.I.C.E. (Società Italiana di Chirurgia Endoscopica e Nuove Tecnologie)
Source: Surg Endosc. 2018 Jan 24;32(6):2986–93. doi: 10.1007/s00464-017-6006-y (PMC5956063; doi:10.1007/s00464-017-6006-y)

TITLE PAGE

WHY LAPAROSCOPISTS MAY OPT FOR THREE DIMENSIONAL VIEW: A SUMMARY OF THE FULL HTA REPORT ON 3D VS 2D LAPAROSCOPY BY S.I.C.E. (Società Italiana di Chirurgia Endoscopica e Nuove Tecnologie)

Authors and affiliations:

Vettoretto Nereo: Surgery, Montichiari - ASST degli Spedali Civili di Brescia, Italy

Foglia Emanuela: Centre for Health Economics, Social and Health Care Management, LIUC-Università Carlo Cattaneo, Castellanza, Italy

Ferrario Lucrezia: Centre for Health Economics, Social and Health Care Management, LIUC-Università Carlo Cattaneo, Castellanza, Italy

Arezzo Alberto: Center for Minimal Invasive Surgery - University of Turin School of Medicine, Italy

Cirocchi Roberto: General and Oncologic Surgery - University of Perugia, Italy

Cocorullo Gianfranco: General and Emergency Surgery - Azienda Ospedaliera Universitaria Policlinico P. Giaccone Palermo, Italy

Currò Giuseppe: General and Oncologic Surgery - University of Messina, Italy

Marchi Domenico: General Surgery - Ospedale Civile di Baggiovara, Italy

Portale Giuseppe: General Surgery - Ospedale di Cittadella, Italy

Gerardi Chiara: IRCCS - Istituto di Ricerche Farmacologiche Mario Negri, Italy

Nocco Umberto: Clinical Engineering - ASST Settelaghi Varese, Italy

Tringali Michele: Direzione Generale Welfare - Regione Lombardia, Italy

Anania Gabriele: General and Thoracic Surgery - University of Ferrara, Italy

Piccoli Micaela: General Surgery - Ospedale Civile di Baggiovara, Italy

Silecchia Gianfranco: General Surgery - Sapienza University of Rome Polo Pontino, Italy

Morino Mario: Center of Minimal Invasive Surgery - University of Turin School of Medicine, Italy

Valeri Andrea: General, Emergency and Minimally Invasive Surgery - Azienda Ospedaliera Universitaria Careggi Firenze, Italy

Lettieri Emauele: Deparment of Management, Economics and Industrial Engineering - Milan Politecnico, Italy and Chalmers University of Technology Centre for Healthcare Improvement, Sweden

Corresponding author: Nereo Vettoretto, Montichiari Surgery, ASST Spedali Civili Brescia, Brescia, Italy. V.le Ciotti 154, 25018, Montichiari (BS), Italy. Tel. +390309963288. E-mail: nereovet@gmail.com

Abstract
BACKGROUND: Three-dimensional view in laparoscopic general, gynaecologic and urologic surgery is an efficient, safe and sustainable innovation. The present paper is an extract taken from a full health technology assessment report on three dimensional vision technology compared with standard two-dimensional laparoscopic systems.
METHODS: A health technology assessment approach was implemented in order to investigate all the economic, social, ethical and organisational implications related to the adoption of the innovative three-dimensional view. With the support of a multidisciplinary team, composed of eight experts working in Italian hospitals and Universities, qualitative and quantitative data were collected, by means of literature evidence, validated questionnaire and self-reported interviews, applying a final MCDA quantitative approach, and considering the dimensions resulting from the EUnetHTA Core Model.
RESULTS: From systematic search of literature , we retrieved the following studies: 9 on general surgery, 35 on gynaecology and urology, both concerning clinical setting. Considering simulated setting we included: 8 studies regarding pitfalls and drawbacks, 44 on teaching, 12 on surgeons' confidence and comfort and 34 on surgeons' performances.
Three-dimensional laparoscopy was shown to have advantages for both the patients and the surgeons, and is confirmed to be a safe, efficacious and sustainable vision technology.
CONCLUSIONS: The objective of the present paper, under the patronage of Italian Society of Endoscopic Surgery, was achieved in that there has now been produced a scientific report, based on a HTA approach, that may be placed in the hands of surgeons and used to support the decision-making process of the health providers.

Key Words: three-dimensional vision, laparoscopy, surgery, health technology assessment, systematic review

Introduction

The introduction of new technologies into the healthcare systems should be guided frequently by qualitative approaches, since the relation between manufactures/suppliers and physicians is not economically sustainable[1] without an empirical and scientific investigation of all the possible benefits and disadvantages related to implementation of the innovation. As recommended by evidence based medicine issues, efficacy and safety information are mandatory for the introduction of a new technology[2]. In recent years, other dimensions have acquired great importance, since a technology assessment could not consist only of cost-effectiveness aspects[3]. This is the reason for the growing attention on health technology assessment (HTA), an instrument useful in guiding the introduction of all healthcare technologies, related to the evaluation of economic, ethical, social, legal, and organisational dimensions consistent with clinical governance tools. Health technology assessment (HTA) is a part of decision-making process in health system and could be considered as a form of policy research and as a source of good evidence and at the meantime that is embedded in further public and professional policy process.

HTA may also be considered as a support in the dialogue between clinicians and healthcare providers[4] and has been verified in the surgical context, where innovation plays a key role; in particular, minimally invasive surgery where, although it requires technological advancement and costly devices to be performed[5], it has proven to be effective in many fields of general, urologic and gynaecologic surgery. One such new technology in laparoscopy uses a new vision method, three-dimensional viewing (3D) that responds to the exigency of improving two-dimensional systems (2D), advancing towards a more realistic, standard and closer to "open surgery" vision. However, while 3D systems are now emerging in the surgical market, as an improvement on 2D systems, they are still not widely disseminated, even though they are used in other non-medical fields.

Moving on from these premises, SICE (Società Italiana di Chirurgia Endoscopica e nuove tecnologie – Italian Society of Endoscopic Surgery and new technologies, which is affiliated to the European Society of Endoscopic Surgery - EAES), has focused its attention on 3D laparoscopic technologies within the Italian surgical setting, by means of a specific survey devoted to SICE affiliates. Sixty-two practices (university and community hospitals, with a private and public ownership) reported that 3D laparoscopy could be a favourable surgical strategy both for surgeons and for patients (65% of responders) and 82% of the surgeons involved in the survey reported that 3D could be considered the “future” of laparoscopy.

As a result, the SICE directory created a multi-disciplinary team (composed of surgeons selected by SICE, operating theatre nurses selected by Italian Association of Operating Theater Nurses - AICO, healthcare sector researchers from LIUC-Cattaneo University, managerial engineers from Milan Politecnico, HTA experts from Lombardy Region, methodology experts and statisticians from Mario Negri Institute for Research), with the aim of producing a scientific report based on a HTA approach.

The objective of the present paper is the evaluation of the 3D technology compared with that of the 2D technology in the settings of general, urologic and gynaecologic surgery, and in various procedures, using a full HTA evaluation.

Methods

To achieve the above-mentioned objective, a health technology assessment was implemented. The evaluation was based on the generally accepted “Core Model” developed by the EUnetHTA Consortium[6], deploying 9 dimensions (version 2.1, April 2015) and considering the related criteria of evaluation derived from the Lombardy Region technologies assessment institutional approach[7].

The following dimensions were examined: general relevance of the pathologies (description of the diseases in which the technology could influence the outcome) and technologies (comparison of the characteristics of the existing 2D and 3D systems), safety issues (morbidity, intraoperative blood loss, safety and drawbacks of surgeons), efficacy, economic impact (activity-based cost analysis and budget impact analysis), equity, ethical and social impact, legal issues, and organisational impact (hospital stay, comfort for the surgeons, operating time, and learning curve). This process was

conducted considering the following setting: general surgery, gynecology, urology, on-going studies, teaching, pitfalls and drawbacks, surgical skills and comfort of the surgeon.

Ad hoc questionnaires were created and administered to a pool of Italian "surgical opinion" leaders (N=8) in order to investigate their perceptions concerning equity, ethical, social, organisational, and legal implications comparing 2D and 3D systems. The questionnaires were structured in accordance with a 7-item Likert scale ranging from -3 (worse impact) to +3 (better impact).

To obtain safety, efficacy and organisational dimensions, a systematic review of the literature evidence was carried out for each clinical or simulated respectively using PICO[9], PRISMA[10] and Cochrane methodologies[11]. PubMed, Scopus and Cochrane databases were systematically searched up to July 2016 for published randomised clinical trials, observational studies, and meta-analyses of the selected setting and interventions.

We used the following terms and also their MESH term(s), if available:3D, three-dimensional, 2D, two dimensional, surgery, laparoscopy. We combined this search with each relevant setting (clinical or simulated) and their specific terms for the following categories: general surgery, gynaecology and urology, surgeons' confidence and comfort, pitfalls and drawbacks, surgeons' performances and teaching.

We also sistematically searched the following registries up to May 2016: www.clinicaltrials.gov, www.clinicaltrialsregister.eu, www.anzctr.org.au, to detect on-going studies aimed to compare 3D vs 2D approaches.

All studies were classified using the table 'Levels of Evidence Oxford Centre for Evidence-Based Medicine 2011' to assess the role of each publication in terms of evidence generated.

After the medical database and registry searches, two reviewers, one surgeon and one methodologist, independently screened abstracts and full texts, and separately proceeded with data extraction. Discrepancies were solved by consensus.

Results

Comparison of the technologies under assessment

A comparative analysis of technologies between 2D and 3D systems used in the Italian market was undertaken using the technical characteristics provided by manufacturers/suppliers. Four different providers of the new 3D technology were contacted and, upon request, made available data on the standard 2D and the new 3D equipment. No significant differences were found between the various 2D systems. All the 3D technologies utilised full-HD 3D (>=1920x1080 pixels) with a 26"-32" monitor. All systems were provided with "light and comfortable" passively polarized glasses and did not require synchronisation with the video images (as with earlier 3D systems[33]). The main characteristics of 2D and 3D systems (visual resolution, images distribution and optics) were comparable and superimposable. The systems consisted of a camera, monitor, light source and image processor: no collateral accessories were considered in the comparative cost and performance analysis. Data on median costs of the laparoscopic columns (based on the industries' sales-catalogues) were 64,774.36 euro for the 2D column and 107,577.54 euro for the 3D column. Data on dissemination of the 3D systems in Italy showed a penetration in Italian surgical units of around 15% (from a total of 876 surgical units, data extracted from an Italian national database[34]). Also considered was that the potential penetration of the new technology could reach approximately 100%, since all surgical units in Italy have a 2D laparoscopic column in their operating theatres.

Efficacy, safety and organisational results: evidence from the literature review

Seven systematic reviews were accomplished, each supported by 8 to 44 papers (comprehending systematic reviews, randomised controlled studies, comparative studies or prospective-retrospective cohort studies) after a selection by titles and abstract, removal of duplicates and exclusion of non-pertinent studies after full-text retrieval, as shown by Prisma flow-diagrams in Appendix 1. Summary of findings and results for each selected study have been drafted and the data summarized for each EuNetHTA dimension.

- *General relevance of the pathology:* the relevance of the health problem was determined by

comparing articles related to different operations for 3D vs 2D laparoscopy in the three settings of general, urologic and gynaecologic surgery. The analysed operations were cholecystectomy, colectomy, adrenalectomy, hepatic resection, pulmonary resection, obesity surgery in the context of general surgery; hysterectomy and pelvic lymphectomy for gynaecological surgery; radical trans-peritoneal and retroperitoneal prostatectomy, pieloplasty, urethroplasty, and radical cystectomy for urologic surgery. A hypothetical representative pool of patients, who had undergone these surgical procedures, was defined as a general case mix of a standard and generic Italian hospital. The hypothesised case mix was derived from a recent survey performed in Campania Region[18]. In this specific setting, in the year 2014, 8,566 patients underwent some kind of general, gynaecologic or urologic surgery. Out of these, 3,544 patients (41.38%) underwent one of the laparoscopic operations described (Table. 1).

-*Evaluation of outcomes*:

Two clinical settings and 4 simulated settings were explored:

- 3D vs 2D in the clinical setting of general surgery: 9 comparative studies (4 randomised controlled studies - RCTs) out of 235 screened papers;
- 3D vs 2D in the clinical setting of gynaecology and urology: 35 comparative studies (1 systematic review -SR and 12 RCTs) out of 667 screened papers;
- 3D vs 2D, pitfalls and drawbacks in simulated settings: 8 comparative studies (5 RCTs) out of 56 screened papers;
- 3D vs 2D, value in teaching for simulated settings: 44 comparative studies (1 SR and 36 RCTs) out of 267 screened papers;
- 3D vs 2D, surgeons' confidence and comfort: 12 comparative studies out of 187 screened papers;
- 3D vs 2D, surgeons' performances in the simulated setting: 34 studies (1 SR and 26 RCTs) out of 149 screened papers.

Significativity was investigated with Excel's descriptive Statistics tool (Microsoft®).

Results in clinical settings concerning morbidity reported a significant difference in urinary continence favouring 3D in radical prostatectomy and cystectomy ($p<0.02$ and 0.05), and a substantial overlap in safety issues for other surgeries.

Haemorrhages were significantly lower for 3D pelvic lymphectomy (38 vs 65ml, $p=0.033$) and radical prostatectomy ($p<0.05$), though similar in other surgical contexts.

The surgeons' safety and comfort in the clinical setting had been examined only by three Italian studies[12,13,14,15] and demonstrated a significant advantage in terms of less visual fatigue and neck pain in 3D laparoscopy. Simulated settings failed to show statistically significant differences in the surgeons' perspective between the two visual systems, even if results seemed inverse favouring the 2D setting but with a discomfort (in particular, related to dizziness and physical discomfort, worse in the 3D setting in 2 out of 19 studies[16,17]) that was described as "tolerable".

The efficacy value used in the analysis was the operating time, related to the two comparators. No significant differences emerged in the clinical setting of general surgery, even if 3D seemed to shorten the median time. This was particularly evident in laparoscopic cholecystectomy and when isolating the subgroup of "non-expert" or novice surgeons, in which the operating time significantly shortened.

To evaluate the organisational impact for general surgery, the differences in operating time were recalculated in terms of median values, considering only the articles with a significant difference between 2D and 3D (Table.2 and Table.3). Tables 2 and 3 show a significant advantage in implementing the 3D technologies, both in general surgery and urology. However, no significant differences were found within the ob-gyn setting. These results, deriving from the literature evidence, were the differential values (if reported) used in the budget impact analysis in order to estimate the overall economic savings assuming the hospital point of view.

-*Hospitalisation*: no significant differences were found between 2D and 3D technologies considering the hospital stay.

-*Surgeons' comfort*: significant differences were observed in depth perception[16,19,20,21,22] and eye-

hand coordination[23,24], favouring the 3D approach.
-*Surgeons' performance*: the experimental setting reported better performances (speed, accuracy) with 3D vision, both in the expert and in the novice surgeons[19,21,25,26,27,28,29]. The reduction in time was related to various tasks at the simulator (peg-transfer, shape and paper cutting, suturing, rope passing, needle capping), all statistically significant, and some studies evidenced a reduction of the error rate[31]. This is particularly significant for the novice surgeons[20,32] who are able to perform difficult tasks more easily and feel more comfortable, and reflects on learning curves with a significant advantage in the performance of the surgical practices overall. Collateral effects (nausea, eye fatigue, visual disturbances) did not show any statistical difference between the two technologies.

Economic dimension

Since no evidence was found concerning the economic impact of 3D technology (from 42 records screened, 27 articles assessed, 27 articles excluded), this dimension was investigated through the implementation of an activity-based cost-analysis[35,36,37] (ABC), considering a 12-month time horizon and assuming the hospital point of view. In particular, the economic evaluation of each patient undergoing a surgical procedure considered both the “surgical pathway” and the “medical pathway” in terms of length of stay, laboratory tests and other diagnostic procedures. Data included direct costs of personnel working in the operating theatre (surgeons, nurses, anaesthetists, auxiliary personnel and technical staff).

The median cost of the surgical pathway (divided in operating room costs and personnel costs) and of medical pathway is summarised in Table 4. There was established the median life of a laparoscopic column to be approximately 8 years that provided a cost per year of 8.096,80 euro for the 2D system, and 13.447,19 euro for the 3D system. A patient's related cost for the two technologies is shown in Table.5. The ABC, in the analysed/optimised context, reported savings ranging from 1.173% to 1.341% per year, in urology and general surgery, while an increase of 0.232% of cost per year was realised in gynaecology.

From a budget impact point of view, the introduction of a new health technology in a specific setting needs a budget impact analysis (BIA) to support the policy-makers' decisions, in different contexts, from health system regulation to the hospital sustainability, both settings with limited economic resources[38]. In this case, laparoscopic operations were compared, presuming that they would all be performed using either 2D or 3D technology, in the same context (high volume hospital with all medical specialties) as analysed previously. Over one year of activity of the three surgical branches, the adoption of a 3D system of vision would lead to an economic saving of 255,035.05 euro (-1,2451%), based on a reduction in the operating time.

To ensure the robustness of the results, a sensitivity analysis was performed, by changing the data on the reduction of the operating time using 3D technology, based on the level of evidence and recommendation of the literature data. In particular, data extracted from articles regarding general surgery, general and gynaecologic surgeries were classified with a strength between 1-2 (randomised controlled studies and metanalysis[12,13,39,40]), and general and gynaecologic surgery classified between 3-4 (comparative non randomised or case series [41,42,43,44,45]). Results of the budget impact sensitivity analysis confirmed the convenience of the 3D systems, with economic advantages ranging from 1.14% to 1.37%.

Organisational, equity, ethical, social and legal impacts: evidence from the professionals' perceptions

These dimensions were investigated with the support of qualitative questionnaires administered to experts in the field of surgery. The results are summarised in Table 6.

With regard to the qualitative assessment of the organisational dimension, it emerged that, over a time-horizon of 12 months, the introduction of the innovative technology required the institutionalisation of specific training courses devoted to the healthcare professionals and support staff directly involved in the procedure. These had a positive impact on both the internal and the purchasing processes. Furthermore, the innovative technology could be considered as the preferable surgical strategy, in particular for its ability to accelerate the learning curve of the operators

involved and its manageability, thus positively affecting the operating theatre time.
From an equity point of view, the adoption of the innovative technology would generate health migration phenomena and would lead to a significant decrease in waiting lists, thus improving access to care in order to meet the citizens' health needs.
With regard to the ethical and social impact, clinicians declared that patients were able to experience a positive impact from the use of the innovative technology due to a decrease in the post-surgical pain procedure and to a lower risk in developing future complications.
The analysis of the legal implications reported that the two technologies under assessment could be considered super-imposable in their measurement, both considering the indication of use for all the surgical procedures and for all the categories of patients, and the presence of authorisations for use.

Discussion and conclusions

The development of laparoscopic surgery, over the past 20 years, has improved the outcomes of patients by reducing surgical trauma, hospital stay, post-operative pain, and performance status. However, laparoscopy is more difficult to perform and to apprehend, mainly due to two-dimensional vision, limited movements and instrumentation, and impaired tactile feedback.
To bypass some of these problems, research has turned to three-dimensional vision, applied *per se* or associated to robotic platforms. To date, there have been few clinical trials as 3D platforms are poorly disseminated in surgical practices, mainly due to cost-containment reasons. Clinical guidelines and consensus conferences are not sufficient in order to validate the introduction of a new surgical technology: the sustainability and the economic impact, associated with the evidence of a clinical improvement (related both to the patients and the operators) requires an in depth examination.
A strategic tool that could overcome the above-mentioned limitations is the health technology assessment (HTA). However, it would require a new common language to be shared between physicians, engineers, managers and healthcare providers.
The present paper may be considered as the first attempt to produce a complete HTA-report within the surgical field, as a multi-disciplinary exercise involving all the professionals, under the patronage of the Italian Society of Endoscopic Surgery and new technologies (SICE), an affiliate of the European Association of Endoscopic Surgery (EAES).
Results of the clinical trials favor 3D vision in terms of blood loss, operative time and hospital stay (though the main results, regarding comfort for the surgeon, have been investigated in simulated settings that have shown a better depth perception, hands-eyes coordination and accuracy). The performances, in particular for novice surgeons, appear to be improved using 3D vision, with faster and more precise resolution of laparoscopic tasks. Sensations of neck and back pain, physical fatigue, nausea and dizziness have different rates between the clinical setting (in which they appear to be worse for 3D vision) and the simulated setting (in which they ameliorate for 3D vision), even if the worse results seem to be associated with earlier 3D systems and lose significance in the later ones.
All this suggested that there was a need of further studies and investigation in order to better point out such drawbacks.
In this view, literature reported 14 ongoing clinical trials related to this topic (clinicaltrials.gov, https://www.clinicaltrialsregister.eu, http://www.anzctr.org.au), 12 of which are RCTs, starting between January 2010 to March 2015 and that are distributed throughout Europe (5), America (2) and Asia (7). Of these, five have been completed but their results have not yet published, seven are still recruiting and two are not yet recruiting. Their research is focused on laparoscopic cholecystectomy (4), hernia repair (2), colectomy (1), pancreatectomy (1), gastric surgery (1), trans-anal endoscopic microsurgery (1), gynaecologic-urologic pelvic surgery (2), and laparoscopy in general (2). Results are expected by 2020.
The multi-dimensional evaluation suggested, as perceived by Italian surgeons in the *ex-ante* survey, some advantages of the implementation of the new technology, without the need of significant economic additional investment for the Healthcare Systems. The economic assessment, in fact,

reports how the implementation of the new technology, in particular if considered whenever a renewal of the laparoscopic instrumentation is programmed, would not require additional investment, thus resulting in a substantial economic neutrality and sustainability. Furthermore, 3D vision could be considered not only a privilege for centres of excellence, but instead the norm, applicable in most different realities of public or private healthcare contexts.
Finally, it should be mentioned that the present study has limitations; for example, the surveys and the consensus were related specifically to surgical experts, without participation of other subjects who nowadays also take part in health decisions, such as patients' associations.

Acknowledgments: Politecnico di Milano, IRCCS-Istituto di Ricerche Farmacologiche Mario Negri, Regione Lombardia, LIUC-Università Cattaneo, Associazione Italiana Ingegneri Clinici, Associazione Infermieri di Camera Operatoria, Federazione Italiana Aziende Sanitarie e Ospedaliere, Bbraun Italia, Conmed Italia, Olympus Italia, Storz Italia. Thanks also to the rest of the scientific committee: Ferdinando Agresta, Valentino Fiscon, Carlo Bergamini, Gaspare Gullotta, Marco Ettore Allaix, Gianandrea Baldazzi, Fabio Cesare Campanile, Giovanni Sgroi, Sarah Molfino, Gian Paolo Zanchi, Tommaso Fontana, Pietro Barbieri, Angelo Iossa, Giuseppe Navarra, Enrico Restini, Luigi Boni, Ugo Elmore, Gianluca Garulli, Mario Guerrieri, Wanda Luisa Petz, Alessandro Puzziello, Simone Vita

Disclosures: Dr. Foglia reports grants from Gilead Sciences Srl, grants from Fondazione Cariplo, non-financial support from ACOI/SIC/SICE/SICU Surgery Societies, personal fees from Formazione Peperosso Srl, outside the submitted work. Dr. Morino reports personal fees from Karl Storz Endoscopie, outside the submitted work. Dr. Tringali reports personal fees and non-financial support from ALTEMS - Catholic University Roma, personal fees and non-financial support from Innovative Medicines Joint Undertaking 2 (IMI2 JU), Bruxelles, personal fees and non-financial support from Ist. Sup. Studi Sanitari G. Cannarella, Roma, personal fees from Universita' Piemonte Orientale, Novara, personal fees from AGENAS (Italian Agency Health Care Quality and Research), Roma, personal fees from MA Provider SrL, Milano, non-financial support from SIFO (Italian Society Hospital Pharmacysts), non-financial support from SHIRE, Milano, non-financial support from Italian Ministry of Health, Roma, non-financial support from ACOI/SIC/SICE/SICU Surgery Societies, outside the submitted work. Dr. Vettoretto reports personal fees from BBraun, outside the submitted work. Drs Anania, Arezzo, Cirocchi, Cocorullo, Currò, Ferrario, Gerardi, Lettieri, Marchi, Nocco, Piccoli, Portale, Silecchia, Valeri have nothing to disclose.

References:

1. Stefanidis D, Fanelli RD, Price R, Richardson W; SAGES Guidelines Committee. SAGES guidelines for the introduction of new technology and techniques. Surg Endosc. 2014 Aug;28(8):2257-71.

2. McPeek B, Mosteller F, McKneally M. Randomized clinical trials in surgery. Int J Technol Assess Health Care. 1989;5(3):317-32.

3. Marshall DA, Hux M. Design and analysis issues for economic analysis alongside clinical trials. Med Care. 2009 Jul;47(7 Suppl 1):S14-20.

4. Institute of Medicine (US) Committee on Quality of Health Care in America. Crossing the Quality Chasm: A New Health System for the 21st Century. Washington (DC): National Academies Press (US); 2001.

5. Chao TE, Mandigo M, Opoku-Anane J, Maine R. Systematic review of laparoscopic surgery in low- and middle-income countries: benefits, challenges, and strategies. Surg Endosc. 2016 Jan;30(1):1-10.

6. EuroScan International Network. A toolkit for the identification and assessment of new and emerging health technologies, 2014, EuroScan International Network: Birmingham

7. Radaelli G, Lettieri E, Masella C, Merlino L, Strada A, Tringali M. Implementation of EUnetHTA core Model® in Lombardia: the VTS framework. Int J Technol Assess Health Care. 2014 Jan;30(1):105-12.

8. Thokala P, Duenas A. Multiple criteria decision analysis for health technology assessment. Value Health. 2012 Dec;15(8):1172-81.

9. Schardt C, Adams MB, Owens T, Keitz S, Fontelo P. Utilization of the PICO framework to improve searching PubMed for clinical questions. BMC Med Inform Decis Mak. 2007 Jun 15;7:16.

10. Liberati A, Altman DG, Tetzlaff J, Mulrow C, Gøtzsche PC, Ioannidis JP, Clarke M, Devereaux PJ, Kleijnen J, Moher D. The PRISMA statement for reporting systematic reviews and meta-analyses of studies that evaluate health care interventions: explanation and elaboration. J Clin Epidemiol. 2009 Oct;62(10):e1-34.

11. Cochrane Handbook for Systematic Reviews of Interventions: Cochrane Book Series Edited by Julian PT Higgins and Sally Green 2008 The Cochrane Collaboration. ISBN: 978-0-470-69951-5

12. Currò G, La Malfa G, Lazzara S, Caizzone A, Fortugno A, Navarra G.Three-Dimensional Versus Two-Dimensional Laparoscopic Cholecystectomy: Is Surgeon Experience Relevant? J Laparoendosc Adv Surg Tech A. 2015 Jul;25(7):566-70.

13. Currò G, La Malfa G, Caizzone A, Rampulla V, Navarra G. Three-Dimensional (3D) Versus Two-Dimensional (2D) Laparoscopic Bariatric Surgery: a Single-Surgeon Prospective Randomized Comparative Study. Obes Surg. 2015 Nov;25(11):2120-4.

14. Currò G, Cogliandolo A, Bartolotta M, Navarra G. Three-Dimensional Versus Two-Dimensional Laparoscopic Right Hemicolectomy. J Laparoendosc Adv Surg Tech A. 2016 Mar;26(3):213-7.

15. Agrusa A, di Buono G, Chianetta D, Sorce V, Citarrella R, Galia M, Vernuccio L, Romano G,

Gulotta G. Three-dimensional (3D) versus two-dimensional (2D) laparoscopic adrenalectomy: A case-control study. Int J Surg. 2016 Apr;28 Suppl1:S114-7.

16. Usta TA, Ozkaynak A, Kovalak E, Ergul E, Naki MM, Kaya E. An assessment of the new generation three-dimensional high definition laparoscopic vision system on surgical skills: a randomized prospective study. Surg Endosc. 2015 Aug;29(8):2305-13.

17. Ko JK, Li RH, Cheung VY. Two-dimensional versus three-dimensional laparoscopy: evaluation of physicians' performance and preference using a pelvic trainer. J Minim Invasive Gynecol. 2015 Mar-Apr;22(3):421-7.

18. Assessorato alla Sanità di Regione Campania (2015), Sorveglianza delle infezioni del sito chirurgico in Campania.

19. Feng X, Morandi A, Boehne M, Imvised T, Ure BM, Kuebler JF, Lacher M. 3-Dimensional (3D) laparoscopy improves operating time in small spaces without impact on hemodynamics and psychomental stress parameters of the surgeon. Surg Endosc. 2015 May;29(5):1231-9.

20. Özsoy M, Kallidonis P, Kyriazis I, Panagopoulos V, Vasilas M, Sakellaropoulos GC, Liatsikos E. Novice surgeons: do they benefit from 3D laparoscopy? Lasers Med Sci. 2015 May;30(4):1325-33.

21. Lusch A, Bucur PL, Menhadji AD, Okhunov Z, Liss MA, Perez-Lanzac A, McDougall EM, Landman J. Evaluation of the impact of three-dimensional vision on laparoscopic performance. J Endourol. 2014 Feb;28(2):261-6.

22. Kong SH, Oh BM, Yoon H, Ahn HS, Lee HJ, Chung SG, Shiraishi N, Kitano S, Yang HK. Comparison of two- and three-dimensional camera systems in laparoscopic performance: a novel 3D system with one camera. Surg Endosc. 2010 May;24(5):1132-43.

23. Chiu CJ, Lobo Prabhu K, Tan-Tam CC, Panton ON, Meneghetti A. Using three-dimensional laparoscopy as a novel training tool for novice trainees compared with two-dimensional laparoscopy. Am J Surg. 2015 May;209(5):824-827.

24. Cicione A, Autorino R, Laguna MP, De Sio M, Micali S, Turna B, Sanchez-Salas R, Quattrone C, Dias E, Mota P, Bianchi G, Damano R, Rassweiler J, Lima E. Three-dimensional Technology Facilitates Surgical Performance of Novice Laparoscopy Surgeons: A Quantitative Assessment on a Porcine Kidney Model. Urology. 2015 Jun;85(6):1252-6.

25. Peitgen K, Walz MV, Walz MV, Holtmann G, Eigler FW. A prospective randomized experimental evaluation of three-dimensional imaging in laparoscopy. Gastrointest Endosc. 1996 Sep;44(3):262-7.

26. van Bergen P, Kunert W, Bessell J, Buess GF. Comparative study of two-dimensional and three-dimensional vision systems for minimally invasive surgery. Surg Endosc. 1998 Jul;12(7):948-54.

27. Tevaearai HT, Mueller XM, von Segesser LK. 3-D vision improves performance in a pelvic trainer. Endoscopy. 2000 Jun;32(6):464-8.

28. Patel HR, Ribal MJ, Arya M, Nauth-Misir R, Joseph JV. Is it worth revisiting laparoscopic three-dimensional visualization? A validated assessment. Urology. 2007 Jul;70(1):47-9.

29. Wilhelm D, Reiser S, Kohn N, Witte M, Leiner U, Mühlbach L, Ruschin D, Reiner W, Feussner H. Comparative evaluation of HD 2D/3D laparoscopic monitors and benchmarking to a theoretically ideal 3D pseudodisplay: even well-experienced laparoscopists perform better with 3D. Surg Endosc. 2014 Aug;28(8):2387-97.

30. Mashiach R, Mezhybovsky V, Nevler A, Gutman M, Ziv A, Khaikin M. Three-dimensional imaging improves surgical skill performance in a laparoscopic test model for both experienced and novice laparoscopic surgeons. Surg Endosc. 2014 Dec;28(12):3489-93.

31. Smith R, Day A, Rockall T, Ballard K, Bailey M, Jourdan I. Advanced stereoscopic projection technology significantly improves novice performance of minimally invasive surgical skills. Surg Endosc. 2012 Jun;26(6):1522-7.

32. Ashraf A, Collins D, Whelan M, O'Sullivan R, Balfe P. Three-dimensional (3D) simulation versus two-dimensional (2D) enhances surgical skills acquisition in standardised laparoscopic tasks: a before and after study. Int J Surg. 2015 Feb;14:12-6.

33. Hanna GB, Drew T, Clinch P, Hunter B, Cuschieri A. Computer-controlled endoscopic performance assessment system. Surg Endosc. 1998 Jul;12(7):997-1000.

34. http://95.110.213.190/PNEed15/

35. Raffish, N and Turney, P (1991). “Glossary of Activity-Based Management”, Cost Management Journal, Fall, 53-63.

36. Drucker P. (1995), “The Information Executives Truly Need”, Harvard Business review, 54-64.

37. Vagnoni E., Potena G. (2003), “L’activity based costing in sanità: il caso dell’ossigeno-terapia”, Mecosan, 47: 149-161.

38. Mauskopf JA, Sullivans SD, Annemans L et al (2007): Principles of good practice for budget impact analysis: report of the ISPOR task force on good research practices-budget impact analysis. Value Health 10:336-47

39. Bilgen K, Ustün M, Karakahya M, Işik S, Sengül S, Cetinkünar S, Küçükpinar TH. Comparison of 3D imaging and 2D imaging for performance time of laparoscopic cholecystectomy. Surg Laparosc Endosc Percutan Tech. 2013 Apr;23(2):180-3.

40. Bove P, Iacovelli V, Celestino F, De Carlo F, Vespasiani G, Finazzi Agrò E. 3D vs 2D laparoscopic radical prostatectomy in organ-confined prostate cancer: comparison of operative data and pentafecta rates: a single cohort study. BMC Urol. 2015 Feb 21;15:12.

41. Velayutham V, Fuks D, Nomi T, Kawaguchi Y, Gayet B. 3D visualization reduces operating time when compared to high-definition 2D in laparoscopic liver resection: a case-matched study. Surg Endosc. 2016 Jan;30(1):147-53.

42. Sahu D, Mathew MJ, Reddy PK. 3D Laparoscopy - Help or Hype; Initial Experience of A Tertiary Health Centre. J Clin Diagn Res. 2014 Jul;8(7):NC01-3.

43.Yang CL, Wang W, Mo LL, Zhang L, Peng GL, Yu ZW, Liu YY, He JX. Short-Term Outcome of Three-Dimensional Versus Two-Dimensional Video-Assisted Thoracic Surgery for Benign

Pulmonary Diseases. Ann Thorac Surg. 2016 Apr;101(4):1297-302.

44. Aykan S, Singhal P, Nguyen DP, Yigit A, Tuken M, Yakut E, Colakerol A, Sulejman S, Semercioz A. Perioperative, pathologic, and early continence outcomes comparing three-dimensional and two-dimensional display systems for laparoscopic radical prostatectomy--a retrospective, single-surgeon study. J Endourol. 2014 May;28(5):539-43.

45. Xu W, Li H, Ji Z, Zhang X, Zhang Y, Xiao H, Liu G. [Comparison of three dimensional and two dimentional laparoscopic pyeloplasty for ureteropelvic junction obstruction]. Zhonghua Wai Ke Za Zhi. 2014 Oct;52(10):771-4.

Table. 1
Case-mix in laparoscopic surgery in a recent audit from a high-volume hospital in Regione Campania

Surgical field	No. of patients (%)
General Surgery	2,567 (72.4%)
Gynaecology	163 (4.6%)
Urology	814 (23.0%)
**Total**	**3,544 (100%)**

Table. 2
Operating times in general surgery

Author	2D	3D	Difference (min)	Difference (%)
Velayutham V, 2016	284	225	-59	-20.77%
Sahu D, 2014	54	40	-14	-25.93%
Currò G, 2015	60	48	-12	-20.00%
Bilgen K, 2013	30	20	-10	-33.33%
Yang CL, 2016	107	86	-21	-19.63%
Currò G, 2015	100	88	-12	-12.00%
			**Median value**	**-21.94%**

Table.3
Operating times in Urology

Fonte	2D	3D	Difference (min)	Difference (%)
Aykan et al., 2014	190	131	-59	-31.05%
Xu et al., 2014	124	106	-18	-14.52%
Bove et al., 2015	241	162	-79	-32.78%
Tang et al., 2016	150.6	133.1	-17.5	-11.62%
			**Median value**	**-22.49%**

Table. 4
Surgical and medical pathway hospital cost

Surgical field	Surgical pathway (devices)	Surgical pathway (human resources)	Medical pathway	Total
General surgery	€ 3,294.57	€ 357.64	€ 2,148.00	€ 5,800.21
Gynaecology	€ 1,505.63	€ 283.79	€ 2,685.00	€ 4,474.42
Urology	€ 3,229.72	€ 320.72	€ 2,416.50	€ 5,966.94

Table.5
Comparative costs per patient per year

Surgical field	No. of patients	2D system	3D system
General Surgery	2,567	€ 1.00	€ 1.67
Gynaecology	163	€ 15.78	€ 26.21
Urology	814	€ 3.16	€ 5.25

Table. 6
Professionals' perception regarding organisational, equity, ethical, social and legal aspects

	Item	3D System
**Organisational aspects**	Additional staff required	0,857
	Staff training	-0,571
	Support staff training	-0,429
	Investment in additional areas	0,571
	Investment in additional equipment	0
	Impact on the purchasing processes	0,286
	Organisational changes	0,429
	Product manageability	1,714
	Improvement in the learning curve	1,714
	**Average Value**	**0,508**
**Equity aspects**	Acceptability of the technology	1,875
	Knowledge of the technology	0
	Hospital waiting list	-0,5
	Accessibility	0,375
	Health migration phenomena	1
	**Average Value**	**0,55**
**Ethical and social aspects**	Patients' autonomy preservation	0,5
	Impact on social costs	0,88
	Impact on post-operative pain	0,5
	Impact on the patients' satisfaction	0,88
	Impact on the development of surgical complications	1,63
	**Average Value**	**0,875**

APPENDIX 1- PRISMA FLOW CHART

PRISMA 2009 Flow Diagram
"3D clinical settings in general surgery"

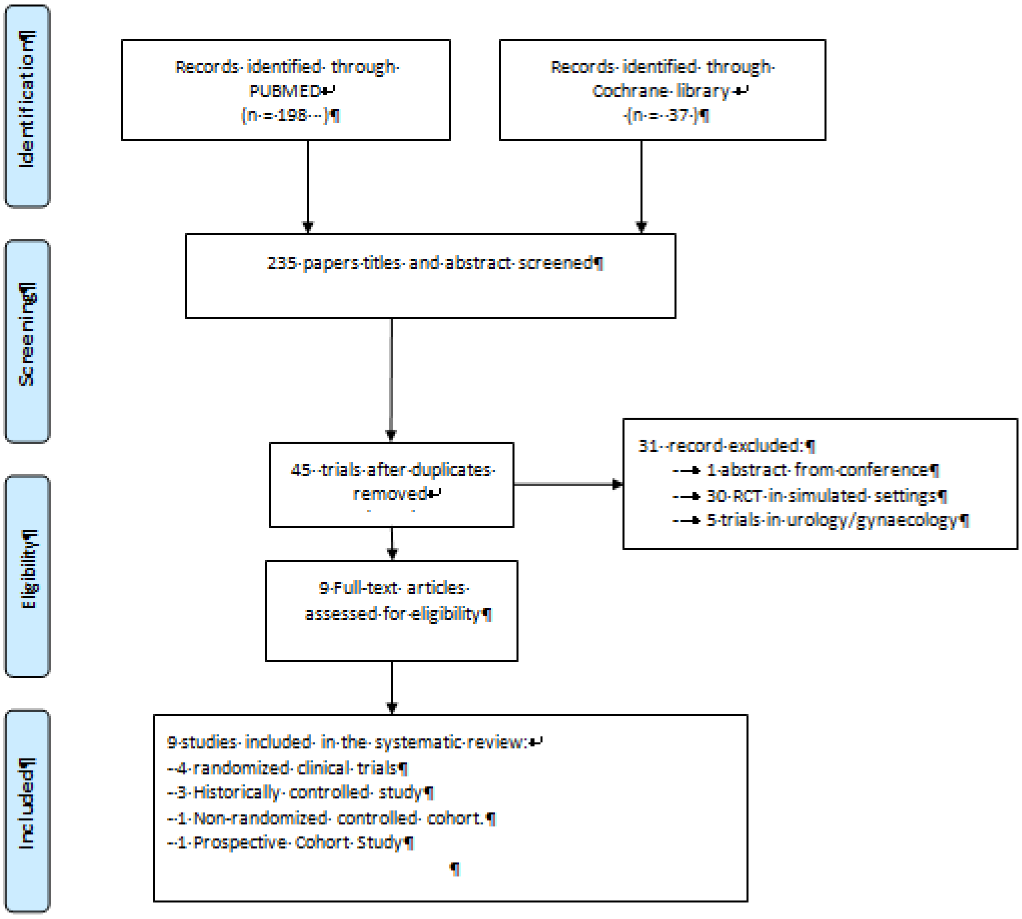

PRISMA 2009 Flow Diagram

"2D versus 3D laparoscopic and robotic clinical settings in gynecological and urological surgery"

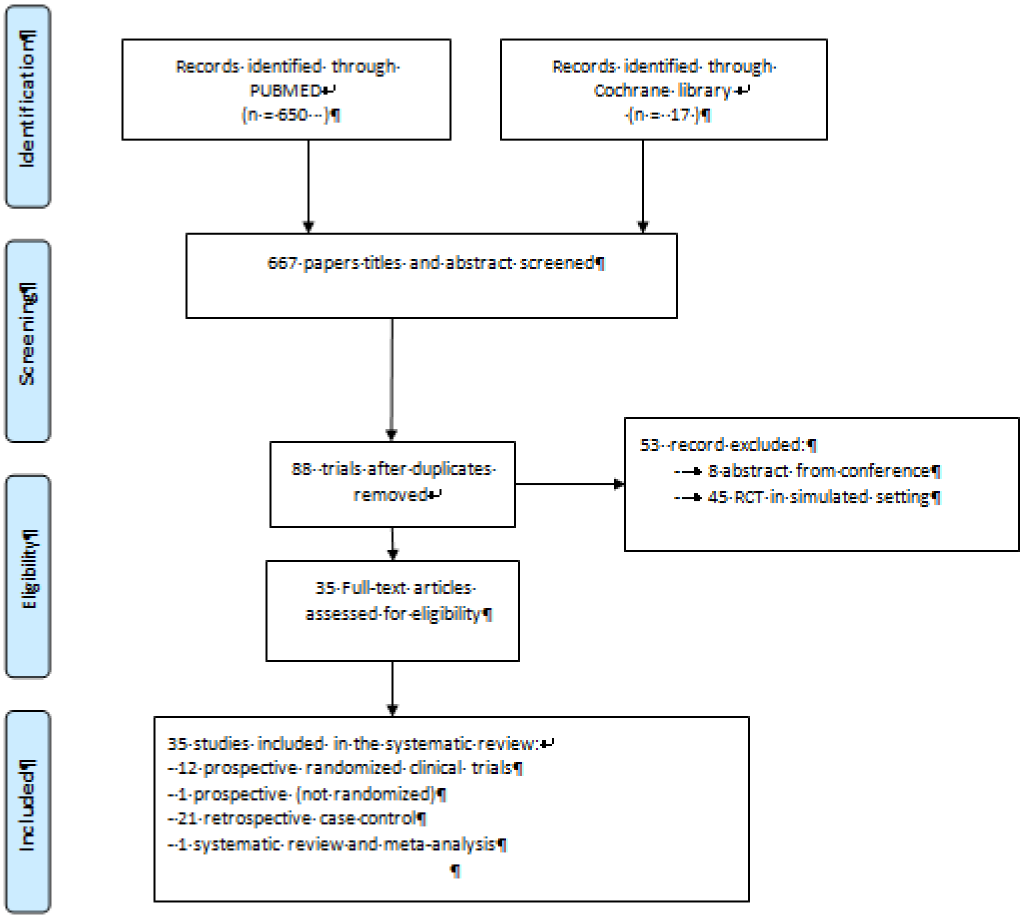

PRISMA 2009 Flow Diagram

"3D simulated setting for teaching"

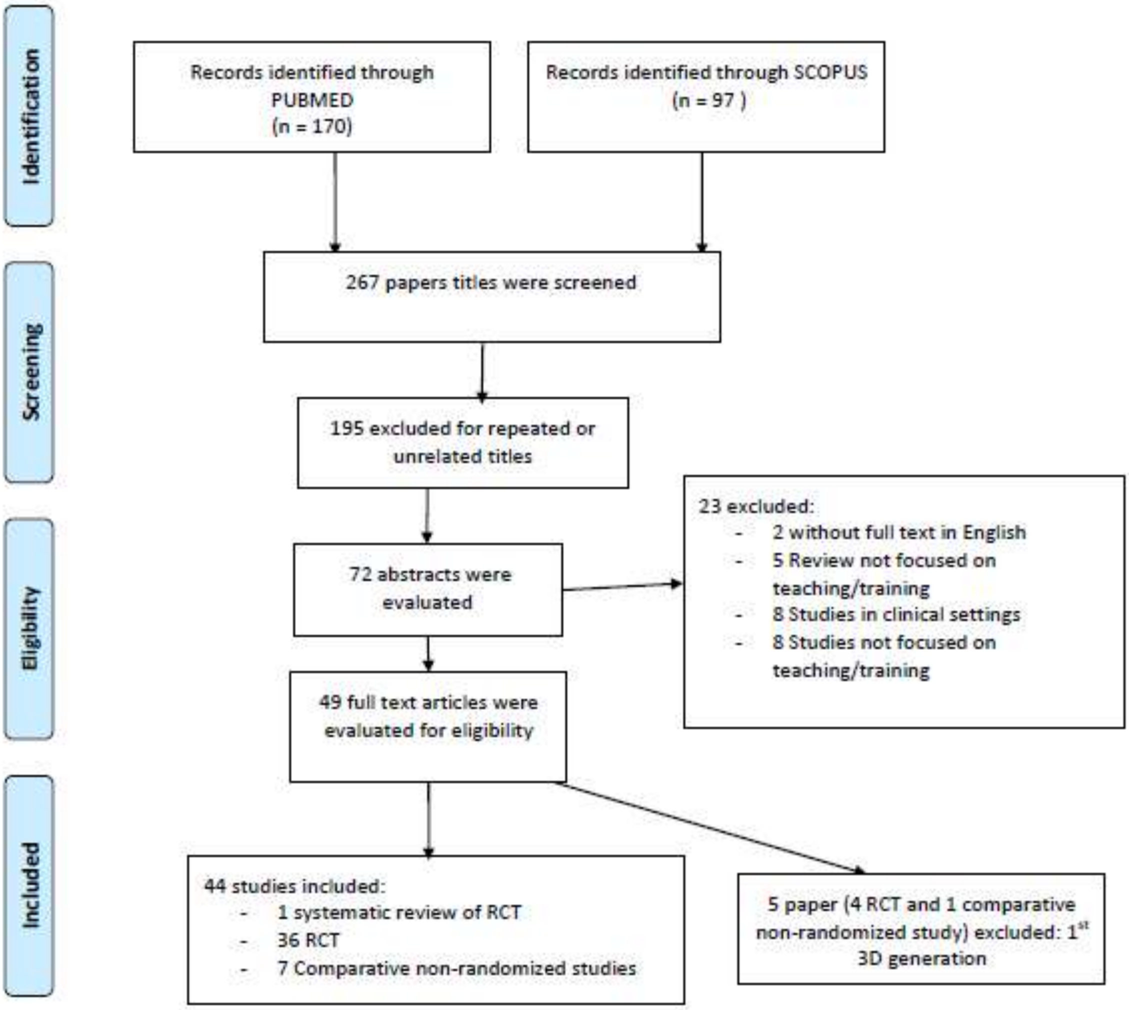

PRISMA 2009 Flow Diagram

"3D simulated setting for surgeon comfort"

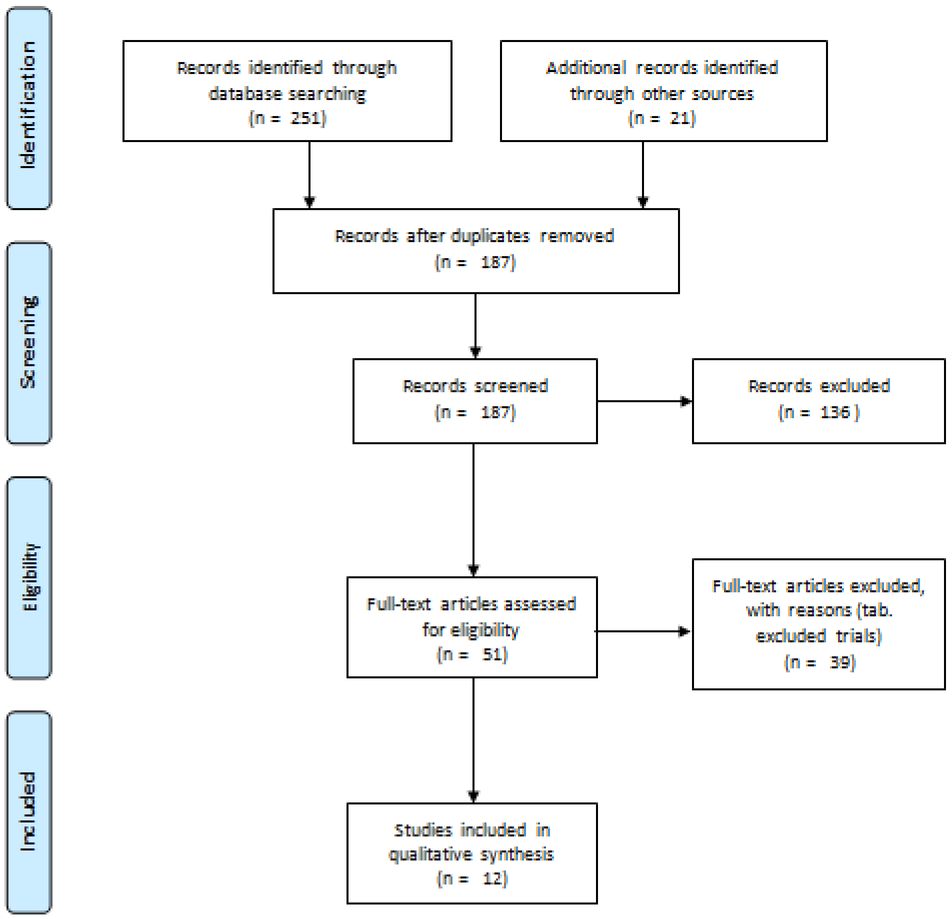

PRISMA 2009 Flow Diagram

"3D simulated setting for surgical Skills"

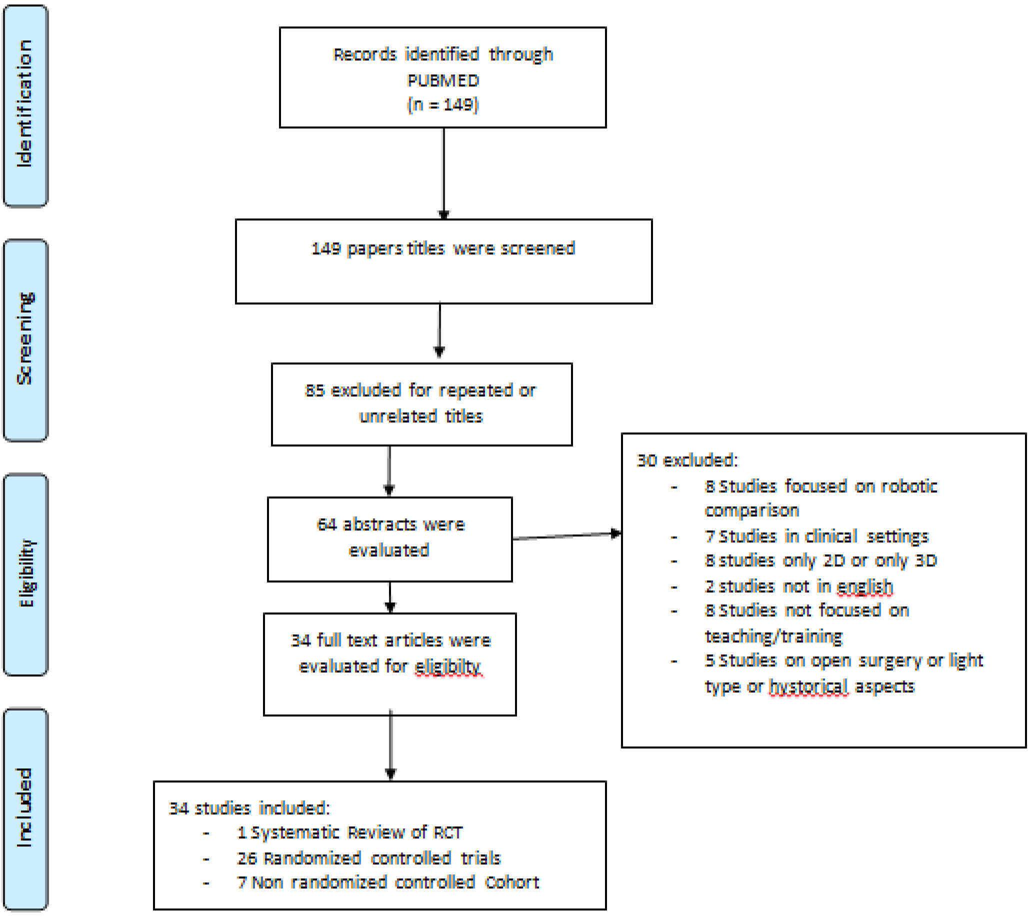

PRISMA 2009 Flow Diagram

"3D simulated setting for pitfalls and drawbacks"

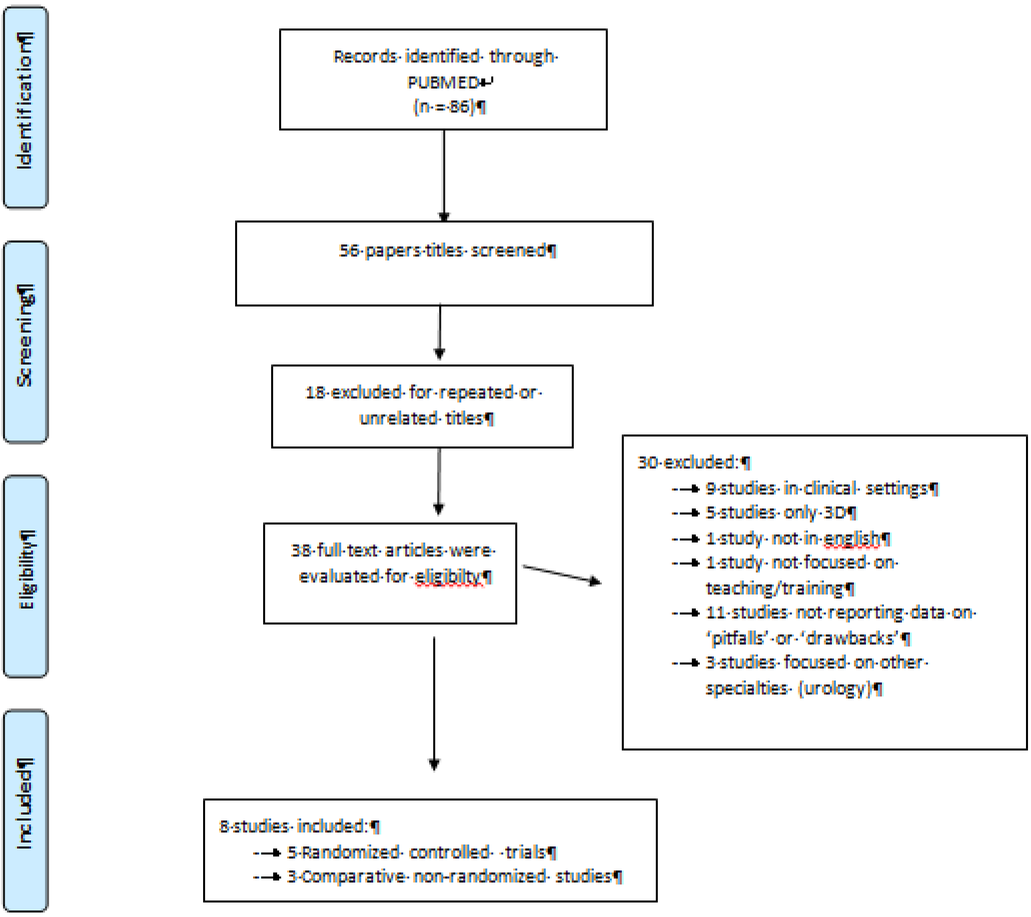

Supplement: Supplementary file 1 — Supplementary material 1 (PDF 378 KB) [file 464_2017_6006_MOESM1_ESM.pdf]
